# Supplementary material for: Chromothripsis during telomere crisis is independent of NHEJ, and consistent with a replicative origin
Source: Genome Res. 2019 May;29(5):737–49. doi: 10.1101/gr.240705.118 (PMC6499312; doi:10.1101/gr.240705.118)
Supplement: Supplemental Material [file supp_gr.240705.118_Supplemental_file_1.zip › contigs/annotated_contigs/DB80/contig.3.DB80_length_483_mean_cov_12.4223602484.docx]

**DB80_length_483_mean_cov_12.4223602484**

```
TTAAACATTGTTGAAAATTTAAGTTCTAACTTAAATGCTGTTTTCAAAAGCGTACTTCACTATTTCTATTCTAATATTCTATCAACAAG
 >chr4:58615881-58616146 - E=6e-148 p=1e-02
TTAATCTGAACACAGCAGTATAAAATGAGACCCTTTTAGGTGAGGCCTTAAGTAACAAAATGGAAGCCTTTACCAAATATGTATTTAAA

CATATTTGGTGAAAAGTGTTCTACTTCAATACAGAGTATATTTCATCAAAATTGCTAAATGTTAACCTTCAAATGTTATTTTTAA|TA|
                                                                                      >c
TATAAAAAGATAATTGAAATATTTCATGGAAATCCTTTTAATTTCACTAATTGAAATTCTATCTGCAATGACTATTCCCTGAGCCTTCT
hr4:58614648-58614746 + E=2e-47
A|TTTTTA|TATACTTCATAAGTGCTTATAATGTTCAAGATACTGTTTAGGATAACTAGGGAATACAGACAAATCTCATTTTCATACAC
  >chr4:58614530-58614658 - E=2e-65
TTATTCTTTAATTTGATTATCTATTTATTCAATAAGTATTTT
```
